# A double-blinded, randomized placebo-controlled trial on the effect of traditional Chinese medicine formula Wuzi Yanzong pill on improving semen qualities in men with suboptimal parameters

**DOI:** 10.1186/s13063-019-3647-2

**Published:** 2019-08-29

**Authors:** Mingpeng Zhao, Carol Pui Shan Chan, Cosy Wing Ching Cheung, Odai Alqawasmeh, Ronald Chi Chiu Wang, Justin Che Yuen Wu, Zhi Xiu Lin, Tin Chiu Li, Jacqueline Pui Wah Chung, Jennifer Sze Man Mak, Tracy Sze Man Law, David Yiu Leung Chan

**Affiliations:** 1 Assisted Reproductive Technology Unit, Department of Obstetrics and Gynaecology, Faculty of Medicine, The Chinese University of Hong Kong, Shatin, Hong Kong; 2 Department of Medicine and Therapeutics, Faculty of Medicine, The Chinese University of Hong Kong, Shatin, Hong Kong; 3 School of Chinese Medicine, The Chinese University of Hong Kong, Shatin, Hong Kong

**Keywords:** Wuzi Yanzong pill, Semen quality, Subfertility, Traditional Chinese medicine, Randomized controlled trial

## Abstract

**Background:**

In Hong Kong, one of six couples is affected by subfertility problems. Male infertility contributes to half of the infertility cases. In male infertility, there is no effective treatment for patients with idiopathic infertility/poor semen parameters. Recent meta-analysis results suggest that a traditional Chinese medicine (TCM) formula — Wuzi Yanzong pill — showed a curative effect on male fertility. However, the heterogeneity of the studies could not draw a definitive conclusion on the therapeutic effect of this formula. The aim of this study is to conduct a well-designed randomized controlled trial to investigate the effect of TCM formula Wuzi Yanzong pill on improving semen qualities in men with suboptimal parameters.

**Methods:**

This study is a double-blinded, randomized placebo-controlled trial conducted in a public hospital in Hong Kong. Participants will be randomized, using computer-generated random numbers, with a 1:1 ratio to either the Wuzi Yanzong pill formula group or the placebo group. Both groups will be administered the drugs for 12 weeks. Participants will have a total of four visits for their semen and blood assessments for a 6-month period, and we will follow up for another 6 months to record their conception outcome. The primary outcome is to compare the total motile sperm count, natural conception rate, and pregnancy outcome to those under placebo treatment. Secondary objectives are sperm functions and assisted reproductive technology outcome.

**Discussion:**

To date, there are no studies using the disclosed Wuzi Yanzong formula or double-blinded, randomized trials. The Wuzi Yanzong TCM formula may provide a good clinical solution for subfertile males for which contemporary western medicine has no cure. Therefore, a well-designed randomized trial for evaluating the effect of Wuzi Yanzong TCM formula is urgently needed.

**Trial registration:**

Chinese Clinical Trial Registry (ChiCTR), ChiCTR-INR-17010790. Registered on 27 February 2017.

Centre for Clinical Research and Biostatistics - Clinical Trials Registry, CUHK_CCRB00548. Registered on 27 February 2017.

**Electronic supplementary material:**

The online version of this article (10.1186/s13063-019-3647-2) contains supplementary material, which is available to authorized users.

## Introduction

### Background and rationale

Subfertility is a common problem around the globe, affecting one in six couples in Hong Kong. Male infertility is responsible for half of the infertility cases in general. Infertility is defined as failure to conceive after 1 year of regular intercourse [1, 2]. Regarding semen quality, both sperm motility and concentration play important roles in achieving pregnancy. Among male infertile patients, there are less than 10% with congenital or genetic abnormalities and 20–30% with pathogenic statuses such as anti-sperm antigen, varicocele, or infection. The rest of the patients (30–50%) have idiopathic infertility (infertility for unknown reasons). This latter group of males presents with no adverse history affecting fertility and has normal anatomical and physiological structure and no endocrine or chromosome abnormalities. Among these males, more than 90% of their infertility cases are due to poor sperm motility, low sperm counts, or both [3].

For those idiopathic infertility males who do not satisfy World Health Organization (WHO) semen parameters, these parameters are categorized with different causes, but the two most important parameters are motility and concentration. Recently, there are reports showing that combining the two parameters as total motile sperm (TMS) count is a better predictive tool for male fertility [4, 5]. In contrast, there is no effective treatment for patients with idiopathic infertility/poor semen parameters.

In recent years, many new approaches have tried to delineate the etiology of idiopathic infertility, including using specific biomarkers, lifestyle analysis, body weight index, and “-omics” approaches, but the aim of developing a promising treatment remains unfulfilled. Testosterone replacement therapy once showed promise as an effective treatment for suboptimal semen quality even though few new testosterone formulations had been developed; however, the efficacies remain uncertain [6]. For this untreatable situation, using traditional Chinese medicine (TCM) may serve as an effective strategy to improve sperm quality in men with suboptimal semen parameters.

Wuzi Yanzong (WZYZ) is a traditional Chinese herbal formula with a long history firstly recorded in a Chinese medical book named Standards for Syndromes and Treatments, written by physician Wang Ken-tang of the Ming Dynasty (A.D. 1368–1644). This traditional Chinese herb formula was well documented in ancient Chinese medical books for its effectiveness. The formula consists of at least five herbs, but there are many variations. The original five-herb form included Fructus Lycii, Semen Cuscutae Chinensis, Fructus Schisandrae Chinensis, Semen Plantaginis, and Fructus Rubi, and this herbal formula has the therapeutic function of tonifying the kidney and supplementing the body essence. In Chinese medicine practice, it is commonly used to treat many kidney deficiency-associated medical conditions such as impotence, infertility, seminal emission, premature ejaculation, low back pain, and urinary incontinence. It has been shown that administration of WZYZ pills can significantly elevate the semen volume and sperm density in infertility patients with low semen counts [7, 8]. However, a large-scale and high-quality randomized placebo-controlled trial on the efficacy of WZYZ pills on semen quality and infertility has never been conducted. It is necessary to conduct a clinical trial to confirm whether WZYZ pills are efficacious in the treatment of patients with poor semen quality.

A recent meta-analysis showed that the WZYZ formula demonstrated a curative effect on male fertility. However, the heterogeneity of the studies did not allow one to draw a definitive conclusion on the therapeutic effect of this formula, mainly due to study problems such as small sample size, unknown randomization methods, bias in the placebo group, and a lack of details on the dosage of the herbal component used. There are no studies using a disclosed WZYZ formula and no double-blinded, randomized trials. An online database search revealed that a well-designed double-blinded, randomized clinical trial with a modest sample size is lacking. The WZYZ TCM formula may provide a good clinical solution for subfertile males for which contemporary western medicine has no cure. The rationale for conducting this randomized controlled trial (RCT) has been further discussed and published in our recent meta-analysis [9].

## Methods

### Aim

The aim of this study is to investigate the therapeutic effect of WZYZ pills on semen quality, sperm functions, and natural conception rate.

### Trial design and setting

This is a prospective, randomized, placebo-controlled, double-blinded, superiority trial with a 1:1 allocation ratio. It is conducted at Assisted Reproductive Technology Unit, Department of Obstetrics and Gynaecology, Faculty of Medicine, The Chinese University of Hong Kong. Our department conducted 892 semen analyses in 2017, in which 273 patients were diagnosed with oligoasthenospermia under the WHO criteria. The Standard Protocol Items: Recommendations for Interventional Trials (SPIRIT) checklist is provided as Additional file 4.

### Eligibility criteria

#### Inclusion criteria

Patients with less than 20 million total progressive motile sperm will be recruited in this study. Since the TMC is so far the most predictive indicator for achieving pregnancy [4, 5], other seminal parameters such as morphology and other physical properties are not included in the criteria.

#### Exclusion criteriaThe exclusion criteria for the study are as follows:

- Male infertility due to one testicle being removed, history of undescended testis, previous chemotherapy, testicular torsion, or any other known abnormalities on reproductive organs
- Patients with azoospermia (indicating structural abnormalities or chromosomal abnormalities)
- Patients with known chromosomal abnormalities
- Patients with normal WHO (5^th^ edition) values for all parameters.

### Interventions

#### TCM WZYZ formula preparations

Traditionally and clinically most Chinese herbal medicines are prepared by decoction in boiling water for hours. In this study, the individual concentrated herbal medicine granules prepared from standardized decoction methods using state-of-the-art concentration technologies according to the Good Manufacturing Practices (GMP) standard will be obtained from PuraPharm® International (H.K.) Ltd. (Hong Kong). The individual concentrated herbal medicine granules will be prepared in sachet form. The modified Wuzi Yanzong pill will be composed of Fructus Lycii 2.4 g, Fructus Rubi 3 g, Semen Cuscutae Chinensis 2.4 g, Semen Plantaginis 2.4 g, and Fructus Schisandrae 2.4 g, plus Radix Rehmanniae Glutinosae Conquitae 3 g, Polygonati Rhizoma 3 g, Cistanches Deserticolae Herba 2 g, Epimedii Herba 2 g, and Cervi Cornus Colla 0.6 g, for a total of 23.2 g divided into two doses per day. For oral administration, the sachets at the designated daily dose are mixed to combine each Chinese medicine. A Certificate for Clinical Trial is not required, as our formula dose package has satisfied the Chinese Medicine Ordinance (Cap. 549 Laws of Hong Kong).

#### Placebo preparation

The placebo is produced by PurePharm International (H.K.) Ltd. with a newly established technology that can mimic the formula’s outlook, color and smell however without any active ingredients and thus no therapeutic effect. The placebo herbs are made indistinguishable in appearance and flavor from the treatment formula. The daily doses of the single form of the herb are packed in individual sachets for easy consumption under the GMP license. This new technology, on one hand, can prevent participants from recognizing being allocated to the placebo group, which could result in a lower compliance rate; on the other hand, it can prevent the potential beneficial effect from using vitamins or clomiphene as a placebo. Patients in the placebo group will consume the same amount of placebo herb as the treatment group.

#### Study process

**Fig. 1:**
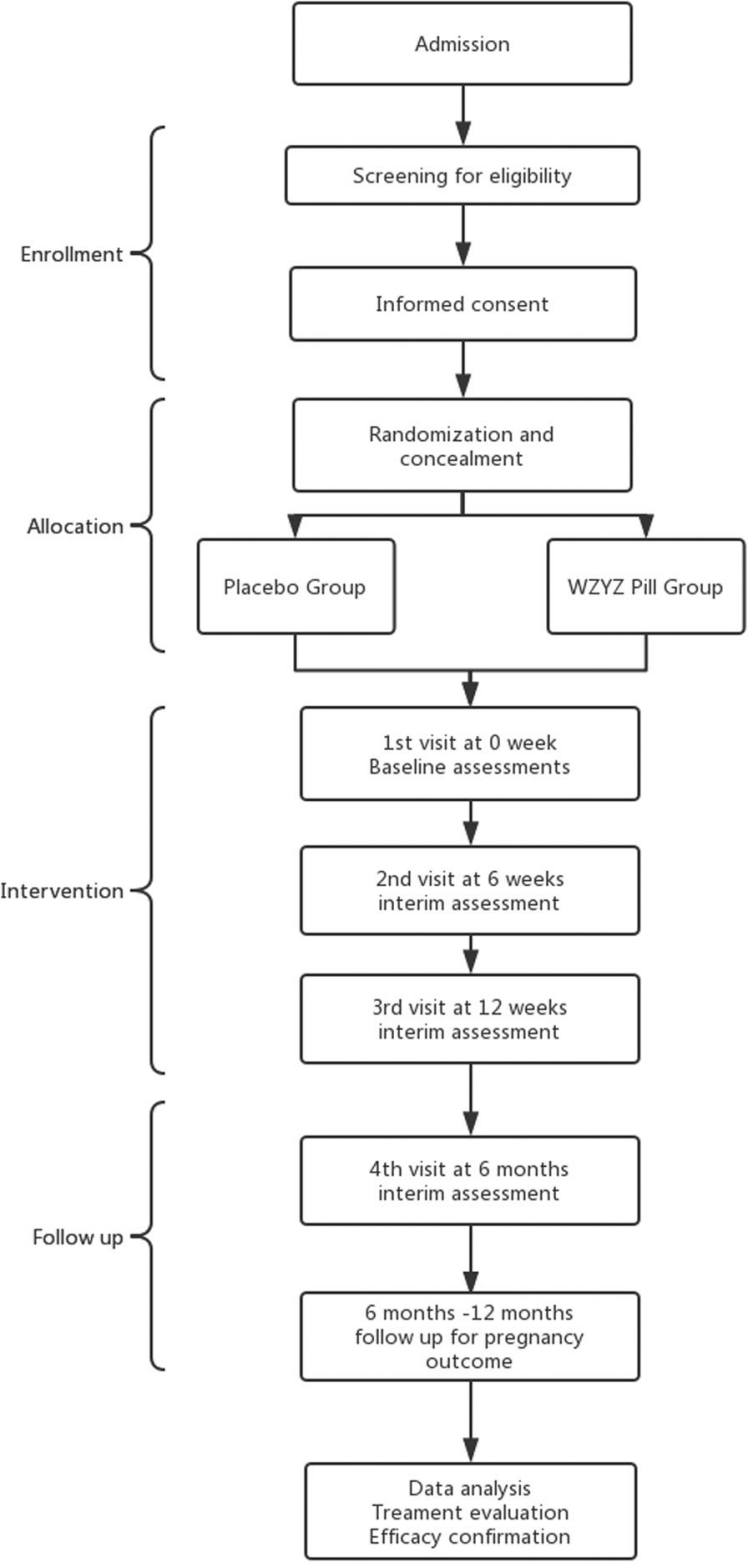
Study flow chart

A semen analysis will be performed for patients in our andrology unit. If the TMS count is less than 20 million per ejaculate, our reproductive medicine practitioner will exam the inclusion and exclusion criteria and consent the patient. After recruitment, the patients are assigned randomly by the TCM team and are divided into the control group taking the placebo and the treatment group taking the WZYZ formula twice a day regularly for a period of 3 months. Semen samples will be taken at the baseline (week 0) and at 6 weeks and 12 weeks to determine if the semen quality has been improved by the formula. An additional semen analysis will be performed 3 months after completion of the treatment. Natural conception and pregnancy outcome will be followed up by our clinical nurse for another 6 months. Compliance will be recorded in the patient diary and by the research nurse via weekly phone calls (90 days of treatment). Patients with less than 63 days of treatment intake will be excluded; the incomplete intake will be considered as failure of compliance too. If the total compliance is less than 70%, then the patient will be considered to have dropped out of the study. The flow chart and study schedule are shown in Fig. 1 and Table 1, respectively. Part of the semen sample will be cryopreserved and separately stored in a − 80 °C freezer for a DNA fragmentation test and other sperm function tests, which will be explained in the “Outcome measurements” sections. A blood sample will also be taken concurrently with the semen samples for hormonal level examination, including routine follicle-stimulating hormone (FSH) and free androgen concentrations.

**Table 1:** Schedule of the study process

Time duration Time point	Study period								
	Recruitment by reproductive practitioner	Allocation by TCM team	Post allocation						
	1st visit	0 week	1–6 weeks	2nd visit^a^	7–12 weeks	3rd visit^a^	3–6 months	4th visit^a^	6–12 months
Enrollment:									
Eligibility screen	X								
Informed consent	X								
Allocation		X							
Interventions									
Administration of investigational product			X		X				
Assessments									
Semen analyses		X		X		X		X	
Blood tests		X				X			
Sperm DNA fragmentation		X		X		X		X	
Sperm cryotolerance		X		X		X		X	
Endocrine-disrupting chemicals test		X		X		X		X	
DNA maturity assay		X		X		X		X	
Liver and renal function assessment		X				X			
Natural conception and pregnancy outcome^b^									X

^a^ The 2nd through 4th visits are scheduled at the end of the time duration

^b^ Natural conception and pregnancy outcome are recorded by phone follow-up

#### Adherence

To enhance the validity of the data, multiple methods will be used to assess medication adherence, including the TCM package count and a phone call follow-up every week asking whether the participant takes the drug accordingly and reasons for non-compliance. Participants will return the unused drugs after 90 days. The unused drugs will be counted and recorded.

#### Concomitant care

The administration of other TCM supplements, as well as nutritional supplements for enhancing sperm parameters, are not permitted for all participants during the first 90 days, as these drugs/supplement may confound the effect of the TCM WZYZ formula.

### Outcome measurements

The primary outcome is to compare the TMS count, natural conception rate, pregnancy outcome, and live birth rate. Secondary objectives are sperm functions and assisted reproductive technology (ART) outcome as well as the safety assessment on the TCM formula.

#### Natural conceptions and pregnancy outcome

Natural pregnancy, ongoing pregnancy, and live birth will be recorded by our team research nurse for a 2-year follow-up after the participant finishes 90 days of drug taking.

#### Semen analyses

A semen analysis will be performed using both manual analyses (by an andrologist) and computer-aided sperm analysis under WHO criteria. The clinical andrologist will have passed the quality assurance test (UK National External Quality Assessment Services [NEQAS]) to make sure he is up to standard. Semen parameters include volume, motility, viability, concentration, morphology, and total sperm count, and another physical status will be measured. A repeat semen analysis will be performed at 3 and 6 months after the treatment to assess the response duration.

#### Blood tests

Hormone FSH and testosterone will be tested, because the levels of the two hormones are closely related to the testis function. Complete blood picture and liver and renal functions tests will also be included to assess the safety of both the TCM formula and placebo.

#### Sperm DNA fragmentation

DNA integrity has been shown to be strongly associated with reduced fertility in males. The increment of DNA fragmentation level reduces fertility in men and affects subsequent embryo development. The level of sperm DNA fragmentation will be analyzed by DNA fragmentation assay to distinguish the effect of the TCM.

#### Sperm cryotolerance

Cryopreservation of sperm is a common clinical procedure for ART treatment; some of the patient’s samples show better sperm cryotolerance than others. Cryotolerance is a test to demonstrate if sperm can tolerate the freeze-thaw procedure; the value is useful to predict the percentage of sperm surviving from cryopreservation for subsequent ART treatment. This test also indirectly provides information on sperm robustness.

#### Endocrine-disrupting chemicals test

Estradiol-like substances in the formula or placebo can possibly reduce the therapeutic effect in males [10, 11]. We will use a patented estrogenic testing method to test the analogs of the WZYZ formula and placebo.

#### DNA maturity assay

Sperm DNA maturity will be tested using aniline blue solution staining. The solution stain histone protein is replaced by protamine during spermatogenesis for dense sperm head compaction. A high histone content indicates poor embryo development and pregnancy outcome.

#### ART outcomes

The ART outcomes include fertilization rate, embryo quality, blastocyst rate, implantation rate, and pregnancy rate. These data will be recorded in the treatment record if the patient receives ART in our hospital. If the patient receives ART elsewhere, the outcomes will be recorded by our team research nurse during a 2-year follow-up.

#### Retention

Once a participant is enrolled, our study investigators and staff will provide written feedback of their semen analysis and liver and kidney function tests. Our research assistant will maintain the participant’s interest in the study through weekly follow-up phone calls, remind the participant of upcoming doctor/data collection appointments, and record the presence of any adverse drug reactions.

### Sample size calculation

Our andrology unit conducted 892 semen analyses in 2017, in which 273 patients were diagnosed with oligoasthenospermia, and the mean of the TMS count of all patients failed to fulfill WHO criteria (5th edition, < 20 million per ejaculate) is 8.08 ± 6.19 million TMS. To detect 100% (double the TMS) improvement by the WZYZ TCM formula and accepting a type I error of 0.05 and a type II error of 0.2, the number of subjects to be recruited into each arm to detect a significant difference is 115 on each arm (Medcalc®, Version 15.6.1). To allow for a dropout/non-compliance rate of 20%, the number of subjects required in each arm is therefore 181, and the total number of subjects required in the study is 286. Therefore, the whole recruitment takes approximately 12–18 months to finish.

### Recruitment

It is estimated that four to five new semen analyses are conducted in our andrology unit per day. We will screen the semen analysis result for a patient’s potential eligibility following the TMS criteria. Once identified, our research assistant will contact the patient, explain the study, and invite him to join it. If the patient is interested, the patient will have another semen analysis and blood drawing to confirm his eligibility. Once the patient fulfills our criteria, our reproductive medicine practitioner will further inform him of our project and ask for his consent.

### Randomization and allocation concealment

As consented patients join the study, they will be randomly assigned in two groups (1:1 ratio) using computer-generated random numbers managed by the Integrative Medicine team. The study medications (TCM WZYZ formula and placebo) will be prepared as similar-looking medications, in the same blisters. The placebo will also mimic the herb’s appearance and smell. Our clinicians who are providing care and the patients themselves will be blinded to the type of treatments for the patients. To achieve high accuracy, the trial will follow the updated guidelines from the Consolidated Standards of Reporting Trials (CONSORT) 2010 Statement [12] and the CONSORT for TCM for reporting our parallel group randomized trials [13]. Unblinding should occur only in exceptional circumstances when problems cannot be solved with ongoing randomization. Unblinding should be performed by an authorized investigator. The investigator should report all unblinding events and their reasons on the corresponding case report form (CRF).

### Quality assurance

A Data Monitoring Committee (DMC) has been established. The DMC is composed of one independent chairman from the Clinical Research Management Office of the Chinese University, one independent member from the Department of Obstetrics and Gynaecology, and one independent member from the School of Chinese Medicine. The responsibilities of the DMC are data monitoring, interim analysis, assessing adverse events and cases with abnormal liver or renal function results after the intervention, reviewing core trial processes and documents, and discussing any amendments to the main study protocol.

### Data management

All information regarding the participant will be carefully recorded on the CRF. All errors will be crossed out and corrected and signed by the corresponding investigator. The study data in the CRF will be entered and coded to a corresponding e-CRF by the double-entry method.

Hard copy CRFs are stored in a separated room and locked in filing cabinets. Only authorized investigators are permitted to access this information. The e-CRFs are stored in a server encrypted using the Advanced Encryption Standard, and only authorized investigators are permitted to access them. The researchers will ensure the confidentiality of sensitive data by minimizing the number of personnel who handle subject data.

### Data analysis

All the data are reported as percentages for nominal data or means ± standard deviation (SD) for continuous data. Patients with less than 70% compliance (i.e., out of 90 days treatment taking the WZYZ formula, less than 63 days of treatment completed) will be excluded. They will be considered as having dropped out and will not be included in the final analysis, but will be included in the intention-totreat analysis. The compliance will be checked by our research staff based on the patient diary and phone calls every 2 weeks. For non-compliance and missing outcomes, we will use two sets of analyses. The full analysis set (FAS) will include all patients who are randomized and will be conducted according to the intention-to-treat principle. The per protocol set will include all patients of the FAS who complete follow-up and do not have serious protocol violations.

For the baseline comparison, the independent Student *t* test or the Mann-Whitney *U* test is used to evaluate the mean or median differences between the placebo and intervention groups for continuous data depending on whether the data are normally distributed, and the chi-squared test is used for frequency comparison. For longitudinal data with a baseline, we will use repeated measures analysis of covariance (ANCOVA) to evaluate the mean difference between and within groups. All the tests are two-tailed, and the significance level is defined as a value of < 0.05.

### Ethical considerations

Ethical approval of the protocol and informed consent forms has been granted from the Clinical Research Ethics Committee (CREC), CREC Reference number 2016.617-T (Additional file 1).

All subjects will be given a detailed explanation of the trial, and their permission will be obtained before they are recruited into the study. A written consent form will be signed by the patient see (Additional files 2 and 3). Semen analysis is a direct and non-invasive method for semen quality evaluation. The TCM remedy is a mild formula with no observable side effects and no known toxicity. We have included liver and renal function tests for each patient at the baseline and at the completion of the WZYZ treatment to assess the safety of the TCM remedy. Blood sample taking will cause extremely mild irritation to the skin, and the infection risk is low.

This study is in compliance with the Declaration of Helsinki and the Guideline for Good Clinical Practice of the International Conference on Harmonisation.

## Discussion

One out of six couples encounters subfertility problems worldwide, and male infertility contributes to half of the cases. Regardless of the cause, there is no effective therapeutic agent available to reverse poor semen quality. Thus, there is an urgent need to explore a potent therapeutic regimen for male fertility. The Wuzi Yanzong formula is a traditional Chinese formula that is effective in improving male sperm quality, but a solid RCT to support this is lacking. This RCT will be conducted by a group of experienced experts from different fields, as the prerequisite to run this project is a group of experts from reproductive medicine, Chinese medicine, and endocrinologists. With our experience in conducting RCTs, this project will demonstrate the compelling clinical effectiveness of the Wuzi Yanzong formula, with the hope that it will lead to the first promising RCT-proven regimen on curing male infertility.

### Trial status

This trial is at Version 1.3, 16/10/2018 (ChiTR). The actual study start date was 27/02/2018; the anticipated study end date is 31/01/2022. The recruitment start date was 27/02/2018; the anticipated recruitment end date is 31/01/2020.

## Additional files

Additional file 1: Ethical approval. (PDF 2090 kb)

Additional file 2: Informed consent form (Chinese version). (PDF 199 kb)

Additional file 3: Informed consent form (English version). (PDF 140 kb)

Additional file 4: SPIRIT 2013 checklist. (DOC 125 kb)

## Data Availability

Not applicable.

## Abbreviations

ART: Assisted reproductive technology
CONSORT: Consolidated Standards of Reporting Trials
CREC: Clinical Research Ethics Committee
CRF: Case report form
DMC: Data Monitoring Committee
FAS: Full analysis set
FSH: Follicle-stimulating hormone
GMP: Good Manufacturing Practices
RCT: Randomized controlled trial
TCM: Traditional Chinese medicine
TMS: Total motile sperm
WHO: World Health Organization
WZYZ: Wuzi Yanzong
