# Supplementary material for: A double-blinded, randomized placebo-controlled trial on the effect of traditional Chinese medicine formula Wuzi Yanzong pill on improving semen qualities in men with suboptimal parameters
Source: Trials. 2019 Aug 29;20:540. doi: 10.1186/s13063-019-3647-2 (PMC6716803; doi:10.1186/s13063-019-3647-2)
Supplement: Supplementary file 1 — Ethical approval. (PDF 2090 kb) [file 13063_2019_3647_MOESM1_ESM.pdf]

香港中文大學醫學院
Faculty Of Medicine
The Chinese University Of Hong Kong

醫院管理局
新界東醫院聯網
Hospital Authority
New Territories East Cluster

Joint Chinese University of Hong Kong-New Territories East Cluster Clinical Research Ethics Committee

香港中文大學-新界東醫院聯網 臨床研究倫理 聯席委員會

8/F, Lui Che Woo Clinical Sciences Building, Prince of Wales Hospital, Shatin, HK
Tel : (852) 2632 3935 / 2144 5926 Fax : (852) 2646 6653 Website : http://www.crec.cuhk.edu.hk

The Joint CUHK-NTEC CREC is an independent committee established by CUHK/NTEC and authorized to perform ethics and scientific review and oversight of clinical studies within the jurisdiction of CUHK/NTEC in accordance with its standard operating procedure and the principles of the Declaration of Helsinki and ICH Good Clinical Practice.

CREC Ref. No.: 2016.617-T

3 FEB '17

To: Prof. Tin Chiu LI
Dept. of Obstetrics & Gynaecology
Prince of Wales Hospital

This notice is issued by the Joint CUHK-NTEC CREC with respect to the application/submission by you, being the principal investigator of the following study at your study site:

- **Study Protocol Title:** A randomized placebo controlled trial on effect of modified Traditional Chinese Medicine (TCM) formula Wuzi Yanzong Pill (WYP, 五子衍宗丸) on improving semen qualities in men with sub-optimal parameters
- **Investigator(s):** Tin Chiu LI, Justine WU, Ronald WANG, Zhixiu LIN, Wing Shan KONG and David Yiu Leung CHAN

In accordance with our standard operating procedure, we have duly performed ethics and scientific review of your application/submission as detailed below:

- **Nature of Your Application/Submission:** ☒ Initial application ☐ Amendments/changes ☐ Others: ☐ Renewal
- **Mode of Review:** ☒ Full review ☐ Expedited review
- **Date of Initial/Renewal Approval:** 03 February 2017
- **Document(s) Reviewed:** See Schedule 1
- **Reviewer(s) :** See Schedule 2

After due review by our reviewer(s), we hereby write to inform you of our decision on your application /submission as follows:

- **Decision:**
  ☒ Application/Submission approved
  ☐ Application/Submission approved with condition(s) (see condition(s) below)
  ☐ Application/Submission approved with remark(s) (see remark(s) below)
  ☐ Application/Submission approved with condition(s) and remark(s) (see condition(s) and remark(s) below)
- **Regular Progress Report(s) Required:** Every 12 months from the date of initial/renewal approval and during the period of the study if required

D David

香港中文大學醫學院
Faculty Of Medicine
The Chinese University Of Hong Kong

醫院管理局
新界東醫院聯網
Hospital Authority
New Territories East Cluster

Joint Chinese University of Hong Kong-New Territories East Cluster Clinical Research Ethics Committee
香港中文大學-新界東醫院聯網 臨床研究倫理 聯席委員會

8/F, Lui Che Woo Clinical Sciences Building, Prince of Wales Hospital, Shatin, HK
Tel : (852) 2632 3935 / 2144 5926 Fax : (852) 2646 6653 Website : http://www.crec.cuhk.edu.hk

3 FEB '17

You, being the principal investigator of the study at your study site, are reminded to comply with our requirements and to maintain communication with us during the period of the study by undertaking the principal investigator's responsibilities including (but not limited to):

- if the study is an industry-sponsored clinical study, submitting to us a copy of the fully executed indemnity agreement satisfying the Hospital Authority's requirement prior to commencement of the study (if it has not been submitted yet);
- observing and complying with all applicable requirements under our standard operating procedure ("IRB/REC SOP"), the Declaration of Helsinki and the ICH GCP (if applicable);
- submitting regular progress report(s) at the required intervals (as specified above) in accordance with the requirements in the IRB/REC SOP;
- not implementing any amendment/change to any approved study document/material without our written approval, except where necessary to eliminate any immediate hazard to the subjects or if an amendment/change is only of an administrative or logistical nature;
- notifying us of any new information that may adversely affect the rights, safety or well-being of the subjects or the proper conduct of the study;
- reporting any deviation from the study protocol or compliance incident that has occurred during the study and may adversely affect the rights, safety or well-being of any subject in accordance with the requirements in the IRB/REC SOP;
- submitting safety reports on all SAEs observed at your study site or SUSARs reported from outside your study site in accordance with the requirements in the IRB/REC SOP; and
- submitting a final report in accordance with the requirements in the IRB/REC SOP upon completion or termination of the study at your study site.

In addition to the above, you are also reminded to observe and comply with other applicable regulatory and management requirements including (but not limited to):

- if required by Hong Kong laws or regulations, obtaining a certificate for clinical trial through the Hong Kong Department of Health and complying with the associated requirements;
- obtaining the necessary consent from the management of your institution/department in accordance with the requirements of your institution/department;
- if required by local laws or regulations at conducting site out of IRB/REC's jurisdiction, obtaining an approval and complying with associated requirements;
- not representing to any third party or in any way likely to mislead any third party forming the view that the approval from the IRB/REC has any extraterritorial effect; and
- with due diligence ensuring your teams, staff, agents or whosoever connected with you to comply with the preceding requirements.

Yours sincerely,

Jenny Ng (Secretary)
for and on behalf of
The Joint CUHK-NTEC CREC

JN/ci

3 FEB '17

Schedule 1
Documents Reviewed

The documents reviewed by with respect to the said application/submission include:

- Research Proposal, Version 1, dated 11 November 2016
- Participant Information and Informed Consent Form, English Version, dated 10 November 2016
- Participant Information and Informed Consent Form, Chinese Version, dated 10 November 2016
- Urgent Contact Card, English and Chinese Version 1, dated 24 January 2017

3 FEB '17

Schedule 2
Reviewers List
Joint CUHK-NTEC Clinical Research Ethics Committee

Title and Name	Occupation	Qualification	Male / Female (M/F)	Study Reviewed by	Present in CREC meeting on 06 Dec 2016
**Chairman:** Prof. Benny C.Y. ZEE	Professor, School of Public Health, CUHK	BSc(Manitoba), MSc(Manitoba), PhD(Pittsburgh)	M	√	√
**Vice/Deputy Chairman:** Dr. Simon K.C. CHAN	Consultant, Department of Anaesthesia and Intensive Care, PWH	MBBS (UNSW), FANZCA(Aust), FHKCA, FHKAM, Dip Pain Mgt(HKCA), MHSM(UNSW)	M	√	√
Prof. Lijia CHEN	Assistant Professor, Department of Ophthalmology Visual Sciences, CUHK	Bachelor of Med (China), Master of Op (China), PhD (CUHK), FRCS (Edin), MRCS (Edin)	M	√	
Prof. Lester AH CRITCHLEY	Professor, Department of Anaesthesia & Intensive Care, CUHK	B MedSci, MB ChB, FFARSCI, FHKAM (Anaesthesiology), MD (Sheffield), GMC (UK) Specialist registrar (UK)	M	√	√
Prof. Alice Pik Shan KONG	Associate Professor, Department of Medicine and Therapeutics, CUHK	MBChB(CUHK), MRCP(UK), FHKCP, FHKAM(Medicine), FRCP(Glasgow)	F		√
Prof. Bonnie Ching Ha KWAN	Associate Professor, Department of Medicine and Therapeutics, PWH, CUHK	MBBS(London), MHKCP, MRCP(UK), FHKCP, FHKAM	F	√	
Prof. Simon LAM	Associate Professor, Department of Paediatrics, CUHK	MBBChir, DCH(HK), DCH(International), MRCPCH, FHKCPaed, FHKAM(Paed)	M		
Prof. Vincent C.T. MOK	Associate Professor, Department of Medicine and Therapeutics, CUHK	MBBS(U Sydney), MRCP(UK), FHKCP, FHKAM, MD(CUHK), FRCP(Edin)	M		
Prof. Cheuk Chun SZETO	Professor, Department of Medicine and Therapeutics, CUHK	MBChB(CUHK), MRCP(UK), FHKCP, FHKAM, DM(CUHK), FRCP(Edin)	M	√	√
Prof. Wai Kwong TANG	Professor, Department of Psychiatry, CUHK	MBChB(CUHK), MD (CUHK), MRCP(UK), FHKCP, FHKAM	M		

Title and Name	Occupation	Qualification	Male / Female (M/F)	Study Reviewed by	Present in CREC meeting on 06 Dec 2016
Dr. Andrea LUK	Resident Specialist, Department of Medicine & Therapeutics, PWH	FHKAM, FHKCP, MHJKCP	F	√	√
Dr. Ernest H.M. MA	Senior Medical Officer, Department of Medicine and Geriatrics, TPH	MBChB, MRCP(UK), Msc Resp Med(Lond), FRCP(Lond, Edin, Ire), FHKCP, FHKAM, MBA, EdD(UTS)	M		√
Dr. Kevin Ka Hang OR	Consultant, Department of Medicine and Geriatrics, SH	MBBS(UNSW), MRCP(UK), FHKCP, FHKAM(Med), FRCP(Edin), BChinMed(HKU)	M		
Dr. Keary ZHOU	Instructor, School of Pharmacy, CUHK	BS(UCLA), PharmD(USC)	F	√	
Mr. Bryan Ping Ho CHUNG	Physiotherapist I, Physiotherapy Department, TPH	BSc in Physiotherapy, MSc in Health Care (Physiotherapy)	M	√	
Ms. Alexandra Dak Wai LO	Chinese Medicine Practitioner, Part-time lecturer and adjunct tutor, Dept of Anatomical and Cellular Pathology, PWH, CUHK	LLB, Hons.(HKU), PCLL(HKU), LLB, Hons.(Peking), LLM (CityU), BchinMed(HKU)	F	√	√
Ms. Emily May Ling CHAN	Retired	DSW(HKPU), RSW Certified Hypnotherapist CISM, UMBC	F	√	√
Mr. Christopher LIU	Executive Director, Liu Chong Hing Investment Ltd.	Bachelor of Arts (Oxford), Master of Arts in Jurisprudence (Oxford)	M		
Mr. Wilson SO	Retired	Bachelor of Social Science, Master of Town and Country Planning	M		
Mr. Ping Hei TAO	Retired	MHRM(MQU), Dip Soc Sci(HKBU)	M	√	√
Mr. Foster YIM	Barrister-at-Law	PCLL(CUHK), JD(CUHK), Msc in Marketing (CUHK),MA in Philosophy (UK), BA (Hons) in Translation (LU)	M		√
