# Supplementary material for: A double-blinded, randomized placebo-controlled trial on the effect of traditional Chinese medicine formula Wuzi Yanzong pill on improving semen qualities in men with suboptimal parameters
Source: Trials. 2019 Aug 29;20:540. doi: 10.1186/s13063-019-3647-2 (PMC6716803; doi:10.1186/s13063-019-3647-2)
Supplement: Supplementary file 2 — Informed consent form (Chinese version). (PDF 199 kb) [file 13063_2019_3647_MOESM2_ESM.pdf]

威爾斯親王醫院
香港中文大學婦產科學系
中國傳統醫藥五子衍宗丸對不佳精液質素影響的隨機對照研究

參與者同意書

我們已知質素不佳的精液可能會導致男性不育。我們仍未知道中國傳統醫藥能否提高精液的質量。這項研究的目的是探討五子衍宗丸對精液質量的影響和其中的機制。

根據世界衛生組織的指引，你的精液樣本被定為不佳。如果你願意參予是項研究，閣下需要:

1. 提供 5-10 毫升血液作賀爾蒙或生化檢測 (如酵素、胺基酸等) 。抽血的過程中會有很輕微的痛楚，及非常小的感染風險。
2. 花 5-10 分鐘的時間填寫問卷。
3. 服用其中一種 (a) 或 (b) 三個月：
   (a) 中藥顆粒: 每種中藥均有一個小包裝。這包括枸杞子 2.4g, 覆盆子 3g, 菟絲子 2.4g,熟地黃 3g,黃精 3g, 肉蓯蓉 2.4g, 仙靈脾 2.4g, 車前子 2.4g, 鹿角膠 1.6g 和 五味子 2.4g。所有中藥顆粒一共 25g，每天分開兩次來服用。這些中藥並沒有副作用。

   或

   (b) 安慰劑：和中藥顆粒(a)的外形完全相同。
4. 在服用完所有中藥顆粒或安慰劑的 6 星期和 3 個月和 6 個月後提供精液樣本。提供精液樣本並沒有任何風險。
5. 我們將會在您完成研究後的 6-12 個月致電給您，詢問有關生育的情況。

您將授權香港中文大學 – 新界東醫院聯網臨床研究倫理聯席委員會查閱有關您涉及研究的資料，作為倫理審查。所有與此項研究有關的資料，均會保密處理。閣下是否參與此項研究，亦不會影響閣下應有的治療。如閣下對此項研究有任何問題及查詢，請致電威爾斯親王醫院婦產科學系輔助生育中心陳耀樑研究助理教授（電話：3505 1764）。若你對參與此研究的權利有疑問，可於辦公時間上午 9 時至下午 5 時 30 分向香港中文大學-新界東醫院聯網 臨床研究倫理 聯席委員會查詢 (電話：3505 3935) 。

若閣下現在充份了解有關是項研究的資料而又決定參與，請閣下在同意書上簽名。您的參與全是自願性質。在簽署同意書後，閣下仍有權隨時放棄參與是項研究。閣下是否參與此項研究，亦不會影響閣下應有的治療。

本人同意參與上述研究。本人明白：

- 參與是項研究純屬自願；
- 本人有權隨時放棄參與是項研究；
- 所有資料會保密處理，並只作研究用途；
- 本人同意*/不同意* 提供臨床樣本及資料，並且數據將儲存 10 年（*請刪除其中一項）。

______________	______________	______________
參與研究者姓名	參與研究者簽名	日期
______________	______________	______________
研究人員姓名	研究人員簽名	日期
