# Supplementary material for: A double-blinded, randomized placebo-controlled trial on the effect of traditional Chinese medicine formula Wuzi Yanzong pill on improving semen qualities in men with suboptimal parameters
Source: Trials. 2019 Aug 29;20:540. doi: 10.1186/s13063-019-3647-2 (PMC6716803; doi:10.1186/s13063-019-3647-2)
Supplement: Supplementary file 3 — Informed consent form (English version). (PDF 140 kb) [file 13063_2019_3647_MOESM3_ESM.pdf]

DEPARTMENT OF OBSTETRICS AND GYNAECOLOGY
THE CHINESE UNIVERSITY HONG KONG
PRINCE OF WALES HOSPITAL

A randomized placebo controlled trial on effect of modified Traditional Chinese Medicine (TCM) formula Wuzi Yanzong Pill (WYP, 五子衍宗丸) on semen qualities in men with sub-optimal semen parameters

Participant information and informed consent form

It is well recognized that poor sperm parameters may result in male infertility. It is uncertain if. Tradition Chinese Medicine (TCM) can help to improve semen quality in men with suboptimal parameters. The aim of this study is to investigate the potential effect of modified Wuzi Yanzong Pill, (五子衍宗丸) on semen quality and the possible mechanism underlying the improvement, if any. We understand that your semen analysis results showed one of the parameters to be sub-optiaml according to WHO standard. Should you decide to participate in the study, you will be asked to:

1. Take 5-10ml blood for hormonal test or other biochemical molecules (such as enzyme or amino acid). Blood taking may cause minimal pain and very minimal risk of infection.
2. Spend 5 -10 minutes to fill in a questionnaire
3. You will be asked to take either one for 3 months:
   (a) Granules of herbal medicine in sachet form dividing to two doses per day (one containing: *Lycii Fructus* 枸杞子 2.4g, *Rubi Fructus* 覆盆子 3g, *Cuscutae Chinensis Semen* 菟絲子 2.4g, *Rehmanniae Glutinosae Conquitae Radix* 熟地黃 3g, *Polygonati Rhizoma* 黃精 3g, *Cistanches Deserticolae Herba* 肉蓯蓉 2.4g, *Epimedii Herba* 仙靈脾 2.4g, *Plantaginis Semen* 車前子 2.4g, *Cornus Cervi Colla* 鹿角膠 1.6g, and *Fructus Schisandrae Chinensis* 五味子2.4g, totally 25g). Upon now, these herbal medicines had no known side effect.
   or
   (b) Placebo granules which will look identical to the active granules.
4. Provide semen sample for analysis after 6 weeks, 3 months and 6 months of taking herbal medicine or placebo granules
5. We will phone contact you about the fertility outcome after 6 -12 months of completion of the study.

You authorized The Joint Chinese University of Hong Kong – New Territories East Cluster Clinical Research Ethics Committee to access your records related to the study for ethics review purpose. All data collected are for the sole purpose of this research and is treated as highly confidential. The study itself will in no way affect your management. If you have any questions and concerns relating to this study, you could contact Prof. LI Tin Chiu, head of reproductive medicine unit, Department of Obstetrics and Gynecology in PWH at 3505 1764. For questions about your rights to participate in this piece of research, please call The Chinese University of Hong Kong -New Territories East Cluster Clinical Research Ethics Committee at telephone no. 3505 3935 during office hours (9am-5:30pm).
If you decide to participate in this study after reading and fully understood the relevant information given to you, please sign the consent form below. Your participation is totally voluntary. After you have signed the consent form, you still have the right to withdraw from the study at any time. Whether you participate in this research or not will not affect the treatment you receive.

Informed consent

I hereby consent to participate in the above-mentioned study. I understand that:
- Participation in the study is voluntary
- I have the right to withdrawal from the study any time.
- All data collected will be kept strictly confidential and will be solely for the purpose of research.
- I agree*/do not agree* for the specimen and data to be stored for up to 10 years future research. (*Please delete one)

______________________	______________________	______________
Subject’s Name	Subject’s Signature	Date
______________________	______________________	______________
Researcher’s Name	Researcher’s Signature	Date
